# Resonant Pressure Micro Sensors Based on Dual Double Ended Tuning Fork Resonators

**DOI:** 10.3390/mi10090560

**Published:** 2019-08-23

**Authors:** Yulan Lu, Sen Zhang, Pengcheng Yan, Yadong Li, Jie Yu, Deyong Chen, Junbo Wang, Bo Xie, Jian Chen

**Affiliations:** 1 State Key Laboratory of Transducer Technology, Institute of Electronics, Chinese Academy of Sciences, Beijing 100190, China; 2 School of Electronics, Electrical and Communication Engineering, University of Chinese Academy of Sciences, Beijing 100049, China

**Keywords:** resonant pressure micro sensor, double ended tuning fork resonator electrostatic excitation, piezoresistive detection

## Abstract

This paper presents resonant pressure micro sensors based on dual double ended tuning fork (DETF) resonators, which are electrostatically excited and piezoresistively detected. In operation, the barometric pressure under measurement bends the pressure sensitive diaphragm functioning as the anchor of DETF resonators and therefore produces eigenfrequency shifts of the resonators. Theoretical analyses and finite element analyses (FEA) were conducted to optimize the key geometries of the DETF resonators with enhanced signal to noise ratios (SNRs). In fabrications, key steps including deep reactive ion etching (DRIE) and anodic bonding were used, where sleeve holes were adopted to form electrical connections, leading to high-efficiency structure layout. Experimental results indicate that the presented micro sensors produced SNRs of 63.70 ± 3.46 dB in the open-loop characterizations and differential sensitivities of 101.3 ± 1.2 Hz/kPa, in the closed-loop characterizations. In addition, pressure cycling tests with a pressure range of 5 to 155 kPa were conducted, revealing that the developed micro sensors demonstrated pressure shifts of 83 ± 2 ppm, pressure hysteresis of 67 ± 3 ppm, and repeatability errors of 39 ± 2 ppm. Thus, the developed resonant pressure micro sensors may potentially function as an enabling tool for barometric pressure measurements.

## 1. Introduction

Resonant pressure micro sensors, which are featured with high resolutions, high accuracies, excellent long-term stabilities, and quasi-digital outputs, are widely used in the fields of industrial control, aerospace aviation, and meteorology [1,2,3,4]. As to excitation and detection of resonators, resonant pressure sensors can work based on electrothermal excitation/piezoresistive detection [5,6], electrostatic excitation/capacitive detection [7,8], electromagnetic excitation/electromagnetic detection [9,10], and electrostatic excitation/piezoresistive detection [11,12]. Compared with other types of resonant pressure sensors, the sensors based on electrostatic excitation/piezoresistive detection are featured with high SNRs and simple structures, and thus this type of resonant pressure sensor is under the intensive studies [13].

In 1992, the first resonant pressure sensor based on electrostatic excitation/piezoresistive detection was reported by Parsons et al [14], where the resonators were not packaged in vacuum and the vibration directions of the resonators were perpendicular to the pressure sensitive diaphragm, which resulted in compromised quality (Q) factors and thus device performances. A laterally driven resonant pressure sensor with vacuum packaging was introduced by Welham et al. in 1999 [15], which was packaged by glass frit bonding but featured low reliability. Later on, Kinnell et al. improved the structure of Welham’s sensor and achieved wafer-level packaging by silicon-silicon bonding in 2009 [16]. However, two silicon-silicon bondings and one anodic bonding were required in the fabrication, which was extremely complicated and suffered from limited yield. In order to simplify the device fabrication process, in our group, silicon on insulator (SOI)-based fabrications and wafer-level vacuum packaging were adopted to form resonant pressure micro sensors based on electrostatic excitation/piezoresistive detection [13,17]. However, the key parameters of the sensor structures were not optimal, which led to lower SNRs and compromised performances.

To address these issues, we have utilized theoretical analyses and FEA simulations to optimize the resonator and sensor structures for resonant pressure micro sensors based on electrostatic excitation/piezoresistive detection to maintain high performance. Besides, simplified and conventional SOI fabrication processes including anodic bonding were employed in the fabrication. The resonant pressure micro sensor was experimentally characterized utilizing open-loop tests, closed-loop tests and even pressure cycling tests to validate the design.

## 2. Design

### 2.1. Sensor Description

**Figure 1:**
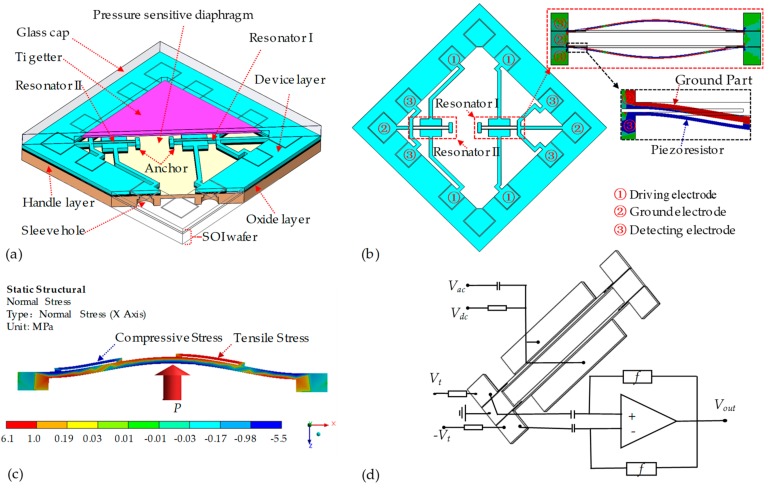
(**a**) Schematic of the proposed resonant pressure micro sensor; (**b**) schematic of the device layer and the resonator of the proposed sensor; (**c**) schematic of axial stresses of the resonators when pressure applied to the pressure sensitive diaphragm; and (**d**) the exciting and detecting circuit of the resonator.

The proposed resonant pressure sensor is shown in Figure 1a. The sensor, with a size of 5 mm × 5 mm × 642 μm, is composed of a glass cap with a cavity (3.26 mm × 3.26 mm × 50 μm) for vacuum packaging and an SOI wafer for barometric pressure sensing which includes a pressure sensitive diaphragm (3.26 mm × 3.26 mm × 100 μm) in the handle layer of the SOI wafer and two DETF resonators (two single 1200 μm × 12 μm × 40 μm beams) in the device layer, where the resonators are coupled to the diaphragm by anchor structures in oxide layer of the SOI layer. In order to increase the sensitivity of the proposed sensor, sleeve holes (the bigger one: Φ 0.5 with a depth of 200 μm, the smaller one: Φ 0.3 with a depth of 100 μm) are used to form electrode vias from the back side of the SOI wafer in the considerations of shrinking down the dimensions of electrode and enlarging the dimensions of pressure sensitive diaphragm. Note that, the two DETF resonators are deployed on the relative central (resonator I) and side (resonator II) areas of the diaphragm along the diagonal direction, respectively, as shown in Figure 1b. Each resonator comprises two driving electrodes, and two double-ended clamped beams. The end of the single beam is divided into a silicon piezoresistor connected to the detecting electrode and a ground part connected to the ground electrode by etching. Like conventional piezoresistive sensors (Chiou et al. [18], Wei et al. [19,20], Li et al. [21]), the location of the maximum stress that marked with blue in the inset of Figure 1b is chosen to arrange the piezoresistor. In particular, the piezoresistor part can be directly used as a strain gauge without having to dope to simplify the fabrication process.

In operations, the deformations of the pressure sensitive diaphragm change the relevant positions of the anchor structures when the barometric pressure under measurement is applied, which would change the axial stresses of corresponding resonators and further leads the eigenfrequency shifts of resonator I to increase and those of resonator II to decrease, as shown in Figure 1c. The corresponding eigenfrequency shifts of the resonator are then detected by a circuit as shown in Figure 1d. A DC voltage *V_dc_* and an AC voltage *V_ac_* are applied to the driving electrodes with a ground voltage to the ground electrode to excite resonator. A pair of DC voltages of ± *V_t_* are used to transform the piezoresistor variances into voltage changes and the corresponding changes of detected voltages are then imported into the amplifier as differential outputs, which further increases the amplitude of output signals.

### 2.2. Sensor Design

#### 2.2.1. Theoretical Analysis of the DETF Resonator

**Figure 2:**
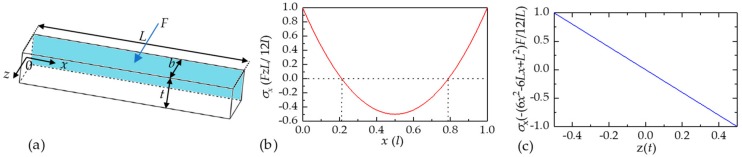
(**a**) The double-ends clamped beam used in theoretical analysis; (**b**) The curve of the axial stresses along the length direction; and (**c**) The curve of the axial stresses along the width direction.

Consider a double-ends clamped beam with width *b*, thickness *t*, and length *L*. A coordinate system is taken with its origin at the center of the cross section of the beam, where the *x*-axis is along the length direction of the beam and *z*-axis is along the width direction, as shown in Figure 2a. In this study, length *L* is much bigger than width *b* and thickness *t*. If the beam was bent to the +*z* direction in the *x-z* plane when a force *F* applied, according to Hooke’s Law, the axial stress along the beam can be expressed as [22]:

(1) $$\sigma (x,z)=-\frac{Fz}{12IL}(6x^{2}-6Lx+L^{2})$$

In this study, the electrostatic force *F* between the driving electrode and the double-ends clamped beam can be expressed as [23]:

(2) $$F=\frac{\partial U}{\partial y}=\frac{1}{2}\frac{\partial C}{\partial y}{\left(V_{dc}+V_{ac}\sin\omega t\right)}^{2}=\frac{1}{2}\varepsilon_{0}\frac{lh}{y^{2}}(V_{dc}{}^{2}+\frac{1}{2}V_{ac}{}^{2}+2V_{dc}V_{ac}\sin\omega t-\frac{1}{2}V_{ac}{}^{2}\cos2\omega t)$$

where, $\varepsilon_{0}$ is dielectric constant in vacuum; *l* is the length of the driving electrode; and *y* is the gap width between the driving electrode and the double-ends clamped beam; *U* is the energy stored in a capacitor.

It can be found that there are tensile stresses in the end areas of the top half part (0< *x* <0.21 *L*, *z* <0) ∪ (0.79 *L* < *x*< *L*, z <0) and compressive stresses in the end areas of the bottom half part (0< *x* <0.21 *L*, *z* >0) ∪ (0.79 *L*< *x*< *L*, z >0) as shown in Figure 2b. In addition, there is a neutral plane (*z* = 0) in the beam and the axial stress distributions in each cross section of beam is symmetric reversely around the neutral plane and linear along the thickness *z* direction from Equation (1), as shown in Figure 2c. To maintain high signal outputs, the piezoresistors are deployed in the tensile stress areas of the double-ends clamped beam.

From Equation (2), the alternating driving force for the vibrations of double-ends clamped beam can be expressed as:

(3) $$\tilde{F}=F_{dc}\sin\omega t$$

where $F_{\mathrm{dc}}{=\varepsilon }_{0}\frac{\mathrm{lh}}{y^{2}}V_{\mathrm{dc}}V_{\mathrm{ac}}$.

The double-ended clamped beam can be equivalent to an *M-K-ξ* mechanical system when driven by the force $\tilde{F}$ [24]. *M* is the equivalent mass of the beam, *K* is the equivalent stiffness of the beam, and *ξ* is the equivalent damping of the system. If the second-order system was subjected to a sinusoidal excitation with a magnitude of $F_{\mathrm{dc}}$, the system response is [25]:

(4) $$h_{\omega }\left(t\right)=\frac{\omega_{n}}{K\sqrt{1-\xi^{2}}}e^{-\xi \omega_{n}t}\sin\omega_{d}t+A\sin\left(\omega t-\phi \right)$$

where:

(5) $$A=\frac{F_{dc}\omega_{n}}{K\sqrt{{\left(\omega_{n}{}^{2}-\omega^{2}\right)}^{2}+4\xi^{2}\omega_{n}{}^{2}\omega^{2}}}$$

(6) $$\phi =\arctan\frac{2\xi \omega \omega_{n}}{\omega_{n}{}^{2}-\omega^{2}}$$

Based on Equation (4), the *M-K-ξ* mechanical system response can be divided into transient (the first item in the right side of the equation) and steady states (the second item in the right side of the equation). Note that, the transient state response decays to 0 rapidly. Meanwhile, the steady state response is a signal with an amplitude of *A* and frequency of ω. Taking the extreme value of the Equation (5), it can be found that the amplitude of *A* is maximum when $\omega =\omega_{n}\sqrt{1-2\xi^{2}}=\omega_{f}$, where $\omega_{f}$ is the eigenfrequency of the beam. When $\omega =\omega_{f}$, the phase difference of the system between driving and the detecting can be calculated as:

(7) $$\phi =\arctan\frac{\sqrt{1-2\xi^{2}}}{\xi }$$

For the system damping *ξ* is very small, the phase difference is closed to 90°, which means that there is a phase difference of 90° between the vibration displacement of the resonant beam and the driving force.

#### 2.2.2. FEA Simulations

**Figure 3:**
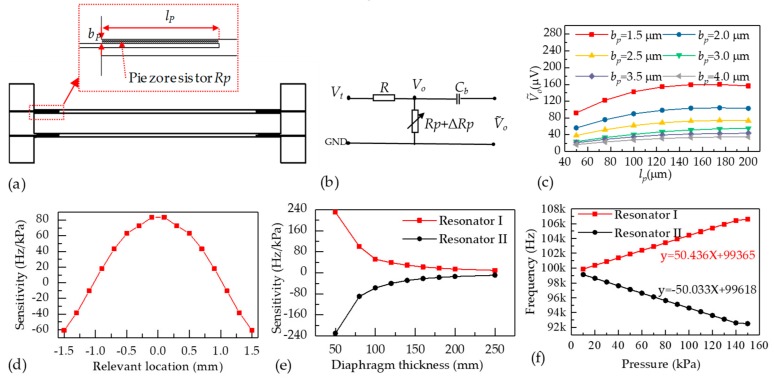
(**a**) The geometry of the DETF resonator using in simulations; (**b**) The electrical equivalent model of the signal detection; (**c**) output ac signal variances to the dimensions of piezoresitor; (**d**) the sensitivity variances to the relevant locations of the resonator with the thickness of the pressure sensitive diaphragm of 100 μm; (**e**) the sensitivity variances to the thicknesses of the pressure sensitive diaphragm with the central beam locating at −0.6 mm and side beam locating at 1.35 mm; and (**f**) the eigenfrequency responses to applied pressure in FEA simulations.

The piezoresistivity model of Structural Mechanics based on COMSOL Multiphysics software was used to calculate the conductivity changes of the silicon material when an additional force is applied. More specifically, tetrahedral elements were used to mesh the geometrical structure of the resonator. The boundary conditions were defined that the electrode structures were constrained. The optimization parameters were the piezoresistor dimensions ($l_{p}\;,b_{p}$ see Figure 3a). Based on the law of resistance, the intrinsic resistance of the piezoresistor $R_{P}$ can be calculated using the intrinsic conductivity of the piezoresistor’s material, and the resistance variances $\Delta R_{P}$ of the piezoresistor can be calculated using the simulation results. To make the simulation results more visual, an equivalent model of the signal detecting is shown in Figure 3b, and the voltage along the piezoresistor can be expressed as:

(8) $$V_{o}=\frac{R_{p}+\Delta R_{P}}{R+R_{p}+\Delta R_{P}}V_{t}$$

where, *R* is the current-limiting resistor. Form Equation (8), the voltage $\tilde{V_{0}}$ generated by the resonator after blocking condenser *C_b_* can be expressed as:

(9) $$\tilde{V_{0}}=\frac{\Delta R_{P}}{R+R_{P}+\Delta R_{P}}V_{t}$$

Thus, the piezoresistor simulations aim to find the optimized relationships between voltage changes of piezoresistor $\tilde{V_{0}}$ and the dimensions of piezoresistor ($l_{p}\;,{\;b}_{p}$). According to Equation (1), the length of piezoresistor should locate within the section of (0, 0.21*l*) and the width of piezoresistor should locate within the section of (−0.5b, 0). In this study, the length of the resonator is 1000 μm and the width of the resonator is 12 μm. Besides, the detect voltage is 5 V and the resistance of the current-limiting resistor is 10 kΩ. Figure 3c shows relationships between $\tilde{V_{0}}$ and the length of piezoresistor $l_{p}$. It can be found that $\tilde{V_{0}}$ is maximum when $l_{p}$ was 150 μm and $b_{p}$ was 1.5 μm.

Besides, pressure sensitivity simulations aim to find the balanced sensitivities by adjusting the thicknesses of the pressure sensitive diaphragm and the locations of the dual resonators to reduce the side effects of temperature disturbances. Multi-models of static structural and modal based on ANSYS software were used to calculate the eigenfrequencies in different applied pressures. In detail, tetrahedral elements were used to mesh the geometrical structures of the sensor. The boundary condition of pressure sensitivity simulations was defined that the bottom of the sensor chip was constrained. Variational pressures of 10 to 150 kPa were used as the loads are applied to the pressure sensitive diaphragm and the outputs of the simulations were the eigenfrequency shifts of the resonators in response to the applied pressures. Eventually, the sensitivities can be worked out using the eigenfrequencies in the pressure range of 10 kPa to 150 kPa.

Figure 3d shows the sensitivity variations in responses to the relevant locations of the resonator with the thickness of pressure sensitive diaphragm of 100 μm. The relationships between the sensitivity and the thickness of pressure sensitive diaphragm with the resonator I located at −0.6 mm and the resonator II located at 1.35 mm are shown in Figure 3e. Taking the matches of sensitivities of dual resonators into consideration, the thickness of pressure sensitive diaphragm was chosen to be 100 μm and the relevant locations of the resonators are chosen to be at −0.6 mm (resonator I) and 1.35 mm (resonator II). The sensitivity of the two resonators is ~ ±50 Hz/ kPa (see Figure 3f).

## 3. Fabrication

**Figure 4:**
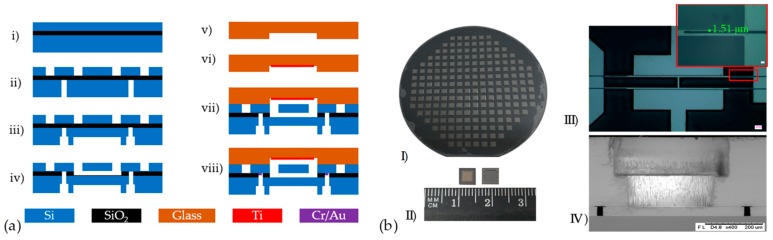
(**a**) The fabrication processes: (**i**) cleaning of the SOI wafer; (**ii**) form the resonators and smaller sleeve holes; (**iii**) form the pressure sensitive diaphragm and the bigger sleeve holes; (**iv**) rRemoving the SiO_2_ beneath the resonators and in sleeve holes; (**v**) rooming the cavity in glass; **vi**) sputtering Ti as getter; (**vii**) anodic bonding and (**viii**) Cr/Au metallization. (**b**) The fabrication results: (**I**) wafer after anodic bonding; (**II**) the front/back views of the sensor chips after dicing; (**III**) picture of resonator; and (**IV**) SEM picture of the sleeve hole.

A 4” customized SOI wafer (device layer: 40 μm, <100> oriented, p-type, resistivity of 0.01 Ω∙cm; oxide layer: 2 μm; and handle layer: 300 μm, <100> oriented, p-type) and a 4” BOROFLOAT® 33 (BF33) glass wafer (Schott, Mainz, Germany) with a thickness of 300 μm were employed. Conventional and simplified micromachined fabrication processes, which includes DRIE, hydrogen fluoride (HF) etching, and anodic bonding, were used to fabricate the sensor chips as shown in Figure 4.

The SOI wafer was dried by pure nitrogen gas after being cleaned by piranha etchant (see Figure 4a(i)). Then, the resonators in the device layer of the SOI wafer patterned by AZ4620 photoresistc (AZ Electronics Materials, Somerville, USA) and the smaller sleeve holes in the handle layer patterned by aluminum (Al)/AZ4620 photoresist composite masks were etched by DRIE (see Figure 4a(ii)). After that, the AZ4620 photoresist in the handle layer of the SOI wafer was cleaned to form the pressure sensitive diaphragm and the bigger sleeve holes by using the left Al mask (see Figure 4a(iii)). Next, the SiO_2_ beneath the resonators and in sleeve holes were etched by HF solution (see Figure 4a(iv)). 

After cleaning the BF33 wafer, the cavities in BF33 glass were formed by HF etching using the patterned gold (Au) film (see Figure 4a(v)). To maintain high Q factors for the resonators, titanium (Ti) film was used as getter to absorb the gas produced during anodic bonding process (see Figure 4a(vi)). Then, anodic bonding was utilized to form vacuum packaged (see Figure 4a(vii)). In the end, the chromium (Cr)/Au film was sputtered to the sleeve holes to form electrical connections (see Figure 4a(viii)).

The fabrication results are shown in Figure 4b, including the wafer after anodic bonding (see Figure 4b(I)), the front/back views of the sensor chips after dicing (see Figure 4b(II)), the picture of resonator (see Figure 4b(III)), and the scanning electron microscopy (SEM) picture of sleeve hole (see Figure 4b(IV)).

## 4. Experimental Characterizations

The resonant pressure sensors were characterized both in open-loop and closed-loop manners. More specifically, an open-loop circuit is used to test the basic characteristics of the two resonators, including the eigenfrequencies, phase differences, Q factors and the SNRs. In addition, a self-oscillating circuit based on closed-loop manner is used to evaluate the performances of the proposed sensor, which include the sensitivities, accuracies, shifts, hysteresis, and repeatability errors. 

**Figure 5:**
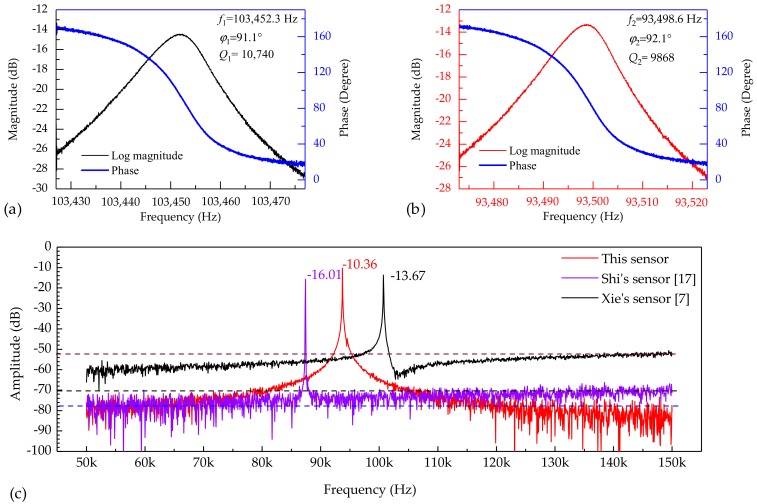
Basic characteristics of the dual resonators: (**a**) and (**b**) the eigenfrequencies, phase shifts, and Q factors of the resonator I and resonator II, respectively; (**c**) the comparison of the SNRs of the sensors based on experimental measurements.

Figure 5a,b show the results of open-loop tests by driving the resonator with bias voltages of 10 V and detecting the piezoresistor variances with a dc voltage of 5 V (with a corresponding detecting current of 0.5 mA) under atmospheric pressure (~ 100 kPa) and room temperature (~ 22 °C). The eigenfrequency of the resonator I was quantified as 103.452 kHz with the phase shift of 91.1° and a Q factor of 10,740 (see Figure 5a). Besides, the eigenfrequency of the resonator II was quantified as 93.499 kHz with a phase shift of 92.1° and a Q factor of 9868 (see Figure 5b). The phase shifts of the two resonators match the solution of Equation (7).

In order to compare the SNRs of the proposed sensor with previously reported counterparts, the outputs of three types of resonant pressure sensors in responses to excitation signals from 50 to 150 kHz with bias voltages of 20 V were measured under atmospheric pressure (~ 100 kPa) and room temperature (~ 22 °C). As shown in Figure 5c, the developed sensor demonstrates a high SNR of 67.64 dB compared with 54.00 dB (Shi’s sensor [17]) and 38.33 dB (Xie’s sensor [7]). Note that, the sensors based on electrostatic excitation/piezoresistive detection shows lower noise levels due to the negligible crosstalk between the detection electrode and driving electrode compared with the sensor based on electrostatic excitation/capacitance detection.

**Figure 6:**
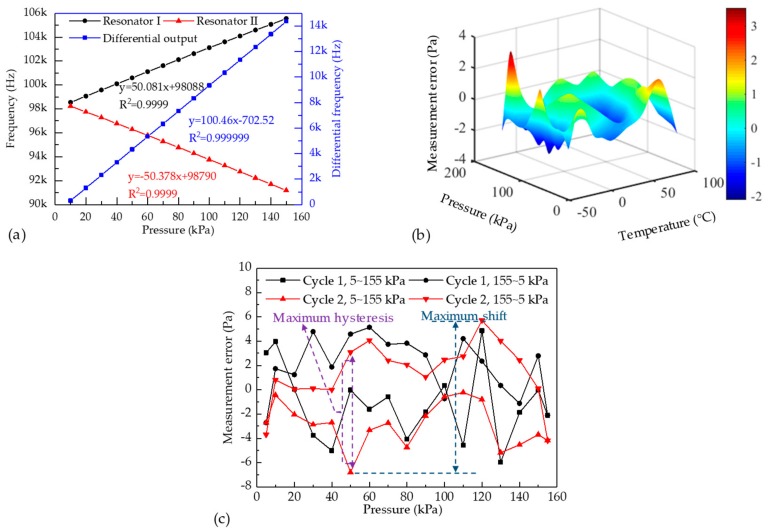
(**a**) Frequency responses to atmospheric pressure under measurement in room temperature; (**b**) the atmospheric pressure measurement errors in the pressure range of 10 to 150 kPa and temperature range of −45 to 85 °C; (**c**) plots of cycling measurement errors to atmospheric pressure under measurement under room temperature.

In addition, the developed sensor was characterized within a pressure range from 10 to 150 kPa and a temperature range from −45 to 85 °C in a closed-loop manner. Figure 6a shows the eigenfrequencies of the two resonators as a function of barometric pressures under measurement at 25 °C, producing sensitivities of +50.1 Hz/kPa for resonator I and −50.4 Hz/kPa for resonator II (with corresponding differential sensitivity of 100.5 Hz/ kPa), which matches the results of FEA simulation. Figure 6b shows the measurement errors of the proposed sensor in pressure range of 10 to 150 kPa and the temperature range of −45 to 85 °C, which produced measurement errors within ±4 Pa with corresponding ±0.01% FS. These two results validated that the developed sensor can achieve accurate barometric pressure measurements.

Furthermore, a pressure cycling test was conducted to further characterize the performance of the developed sensor. In this test, the barometric pressure under measurement was increased from 5 kPa to 155 kPa firstly, and then decreased back to 5 kPa at room temperature. Figure 6c shows the measurement errors of the pressure sensor in the pressure cycling test. The measurement errors to the standard reference pressure were within 0.01% FS for the proposed resonant pressure sensor. The maximum shift of the developed sensor is recorded as 12 Pa with corresponding 83 ppm. Besides, the maximum hysteresis of the sensors is 10 Pa with corresponding 66 ppm.

The repeatability error can also be obtained from Figure 6c. According to the Chinese standard of GB/T 15478-2015, the repeatability error is defined by:

(10) $$\xi_{R}=\frac{\lambda S}{Y_{FS}}\times 100\%$$

where, *Y_FS_* is the full scale of pressure measurement; *S* is the standard deviation of the measurement errors; *λ* is coverage factor. In this work, the full scale of pressure measurement *Y_FS_*, the standard deviation of the measurement errors *S*, and the coverage factor *λ* are 150 kPa, 1.5 Pa and 3, respectively. Therefore, the repeatability error of the proposed pressure sensor is 39 ppm. The results further validated the high performance of the developed sensor.

Table 1 shows the characterized results of four samples of this type of sensor. The four samples exhibit equivalent performances whether in the open-loop tests or in the closed-loop tests, which shows the rationality of the sensor design.

Table 2 compares the key performances of the developed sensors in this study with previously reported counterparts. In comparison to previous resonant pressure micro sensors based on electrostatic excitation and capacitive detection (e.g., Xie [7], Sun [8]), the sensors developed in this study were featured with lower crosstalk and thus higher SNRs. In comparison to the counterparts based on electrostatic excitation and piezoresistive detection (e.g., Shi [13], Shi [17]), the sensor developed in this study demonstrated higher SNRs, higher differential sensitivities, wider working temperature ranges and lower device dimensions due to structure optimization. In addition, the DETF resonators can operate in the anti-phase mode, which ensures that the proposed sensors demonstrate higher pressure cycling performances (e.g., shift, hysteresis and repeatability error) than what was reported in [8].

## 5. Conclusions

Resonant pressure sensors based on dual DETF resonators are presented in this paper. Theoretical analyses and FEA simulations are performed to optimize the output signals of the resonator and the sensitivities of the developed sensors. The presented sensors are fabricated based on conventional and simplified fabrication processes, where a sleeve holes design was adopted to enlarge the dimensions of the pressure sensitive diaphragm and further increase the sensitivity of the sensor. The experimental results show that the proposed resonant pressure sensors exhibit high SNRs of 63.70±3.46 dB, which are ~10 dB higher than previously reported counterparts. The sensitivities of the two resonators of the developed sensors are ±50 Hz/kPa, which matches the simulation results. Further characterizations indicate that the developed sensors demonstrate low measurement errors within 0.01% FS in the pressure range of 10 to 150 kPa and temperature range of −45 to 85°C, low pressure shifts of 83±2 ppm, low pressure hysteresis of 66 ± 3 ppm, and low repeatability errors of 39 ± 2 ppm.

## Figures and Tables

**Table 1:** Performance comparisons of this type sensors.

Characteristics		Sample 1	Sample 2	Sample 3	Sample 4	Average
Q factor	Resonator I Resonator II	10,740 9868	10,438 9765	10,148 9641	10,713 9683	10,510 ± 240 9739 ± 87
Sensitivity (Hz/kPa)	Resonator I Resonator II	+50.1 −50.4	+51.2 −52.1	+51.1 −49.3	+50.8 −50.2	+50.8 ± 0.4 −50.5 ± 1.0
SNR		67.64 dB	58.53 dB	62.75 dB	65.88 dB	63.70 ± 3.46 dB
Accuracy		0.01% FS	0.01% FS	0.01% FS	0.01% FS	0.01% FS
Shift		83 ppm	86 ppm	80 ppm	82 ppm	83 ± 2 ppm
Hysteresis		66 ppm	70 ppm	62 ppm	68 ppm	67 ± 3 ppm
Repeatability error		39 ppm	36 ppm	40 ppm	42 ppm	39 ± 2 ppm

**Table 2:** Comparisons of the sensor performances.

Characteristics	Xie [7]	Sun [8]	Shi [13]	Shi [17]	These Sensors
SNR	38.33 dB	30 dB	/	54.00 dB	63.70 dB
Sensitivity	166 Hz/kPa	29 Hz/kPa	79 Hz/kPa	48 Hz/kPa	101 Hz/kPa
Sensor dimension	9 × 9 mm	/	9 × 9 mm	7 × 7 mm	5 × 5 mm
Q factor	11,000	10,000	10,000	17,000	10,000
Temperature range	−40~70 °C	−40~80 °C	−35~85 °C	−35~85 °C	−45~85 °C
Accuracy	0.02% FS	0.05% FS	0.01% FS	0.01% FS	0.01% FS
Shift	/	/	/	/	83 ppm
Hysteresis	/	500 ppm	/	/	67 ppm
Repeatability error	/	100 ppm	/	/	39 ppm
